# Partial Paralysis of the Soft Palate Following Removal of Tonsils and Adenoids

**Published:** 1915-09

**Authors:** Dunbar Roy

**Affiliations:** Grand Opera House, Atlanta, Ga.


### PARTIAL PARALYSIS OF THE SOFT PALATE FOLLOWING REMOVAL OF TONSILS AND ADENOIDS. OBSERVATIONS ON THE TONSIL AND ADENOID OPERATION.

By Dunbar Roy, M. D., Atlanta, Ga.

Quite a little has been written within the last two years concerning some of the ill effects following the radical removal of the faucial tonsils. The following case came under my observation within the last year.

J. H.—male—age 4½ years, was operated upon in the hospital on May 20th, 1913, for the removal of tonsils and adenoid tissue in the naso-pharynx. Complete removal was accomplished without trouble and with less traumatism than usual. Two days later the patient was out playing, with no signs of bad after effects. The throat was not painful on deglutition and there was but a slight amount of exudate in the tonsillar cavities.

On June 1st, ten days after the operation, he was brought to my office with the following history: Two days previous the child showed a temperature of 101 degrees, some malaise and some inability to articulate distinctly. In swallowing liquids, some of these came back through his nose. Two days before noticing these symptoms the patient had been allowed to eat excessively at a Sunday dinner.

Examination showed the pharynx and naso-pharynx in a good condition. There was only slight signs of inflammation. The tonsils had been thoroughly extirpated and both pillars appeared normal. No pain. The tongue was white and coated. When the patient talked there were all the signs of paresis of the soft palate just as is frequently seen after diphtheria.

The patient was ordered a grain and one-half of calomel to be followed with an enema. He was placed on small doses of strychnia and light diet. June 4th, temperature continues 100½ pretty constantly. The laxative was followed by considerable offensive excretion from the bowels. The uvula looks a little thickened. His physician saw the case with me and gave it as his opinion that the paresis was due to intestinal toxemia. June 6th. This was the first day that the temperature has been normal. Strychnia continued internally. Patient seems well other than the difficulty in speech. The naso-pharnyx was irrigated with an alkaline solution and then touched with a solution of argyrol. Spray through the nose was used at home. June 13th. Patient has had no further temperature. Is apparently perfectly well except the voice. Is having massage in addition to the strychnia. Can swallow without the liquid returning through the nose. Soft palate still has a leathery look. At this time the mother took the child on a visit to her old home in Savannah, Ga. While there she consulted a pediatrist who concluded that the case was one of chorea such as has been described by Osler in his text-book. The same line of treatment was continued. June 24th. Patient was seen on this date immediately after his return. Looks well but talks almost the same. The patient continued to improve but it was nearly three weeks later before the voic was practically normal. There has been no further trouble. The condition therefore lasted for nearly two months.

The ill effects upon the throat following the radical removal of tonsils and adenoids have been commented upon during the last two years by many observers.

Dr. Virginius Dabney read a paper before the American Laryngological Society in 1913 as to the sequela of the radical removal of tonsils based on the observation of 200 cases and at the same meeting Dr. Payson Clark read a similar paper on the results seen in the Massachusetts General Hospital. The question of paralysis or paresis of the soft palate as one of the sequelae of this operation was not mentioned by either of these writers which leads me to the conclusion that such was not observed. In addition to the report from these observers the literature for the last few years contains many others in none of which has this sequela been mentioned.

Inexperienced operators and operators obsessed with the idea of radicalism have caused much damage to throats which would otherwise have been better had they never been touched. Operations for the removal of tonsils and adenoids should not be looked upon as a simple procedure but should have the same thought and care as any other major surgical operation. To operate hurriedly with the possibility of damaging the soft palate and the faucial pillars is inexcusable. To operate entirely by the sense of touch as advocated by some is certainly to be condemned. Every good operator has his own method which is perfectly proper and justifiable provided the technique accomplishes the results desired. Some tonsils can be removed in their capsule by almost any technique because they are large and pedunculated. In such cases there is no better instrument than McKenzie's tonsillotome and it will always do the work thoroughly. In the submerged variety some dissection followed by the use of the snare will certainly give the best results although there will be more reaction.

Different operators accomplish the same results by different techniques. The operator should find the one technique which gives him the best results, meaning by that which leaves the throat in as normal condition as possible and adhere to the same in all of his operations.

Because one man writes about a certain technique which appears different is no reason why the good operator should forsake his own and try another.

Like the observation of others, I have also seen within the last few years various mutilations of the pharynx caused by the so-called tonsillectomies. It is by no means uncommon in such cases to find the anterior and posterior pillars all united into one flat cicatrix and in many cases marked restricted movements of the palate.

The stripping of mucous membrane from the soft palate has also been seen sometimes in conjunction with the total ablation of the uvula. This is always due to the imperfect or faulty separation of the posterior pillar from the tonsils. The accidental removal of the uvula does not occur from the uvula being caught into the snare as a distinct organ, but comes from the posterior pillar not being entirely freed from the tonsils, in this way the palatal mucous membrane and the uvula are drawn into the wire snare.

Mukuen in a most excellent and timely article has called attention to the various defects in speech which may follow the faulty removal of faucial tonsils.

Injury to the muscles of the soft palate, the result of a radical removal of adenoids, has never been considered so far as one may gather from the various text-books and articles bearing upon this subject. The case here reported is the only one of its kind I have ever seen and the fact that a tonsillectomy was also performed keeps us from saying just which operation was the cause of the paresis. On the other hand, there are arguments on the other side, namely, that the paresis was accidental and entirely independent of the operation. This is especially true since cases of paresis of the soft palate have been reported as being one type of chorea. The fact that the paresis occurred ten days after operation does not entirely exclude the condition as standing in the light of a propter hoc. In the light of this experience, I am firmly convinced that operators too frequently use an unnecessary amount of force and traumatism in the removal of adenoids. In the very young subject, say from 3 to 6 years of age, the nasopharynx is very small and it is very easy to stretch the soft palate with any instrument used. The fact is we can unintentionally do the same thing by a digital examination in these cases for diagnosis. In the case reported I used a small size Brandege's forceps for the removal of the adenoids. Since that time I have abandoned forceps in this operation because I am convinced that in opening forceps in the naso-pharynx of small children, there is every opportunity for a traumatism of the soft palate to occur. Such abrasions on the posterior surface can easily be the portals of entrance for various micro-organisms which can always be found in a naso-pharynx filled with adenoid tissue. I think also that operators are too prone to use large size instruments for this operation which can be better accomplished by smaller ones. The LaForce adenotome is one of our best instruments but this too is very apt to be used too large. The instrument without the large anterior bulbous portion is much the better instrument since this latter frequently causes the stretching and traumatism of the soft palate such as we wish to avoid. Small Gottstein currettes following the use of this instrument is to me the ideal operation. The method which some have of using the finger to smooth and remove any particles which may be left, is a thoroughly proper procedure provided the finger is wrapped with sterile gauze. Even with this procedure great care must be exercised in not using too much force.

In the case here reported my own opinion is that the paresis followed the adenoid portion of the operation and that the infection was the result of a traumatism on the posterior service of the soft palate which to my mind was manifested by the thick leathery condition found ten days after the operation. Every operator has seen how severe reactions following a tonsil operation will vary in different individuals. These reactions are some times very severe and for days the patient has an exceedingly uncomfortable throat. Under such circumstances we cannot be surprised that the normal function of the palatal muscles will be somewhat inhibited.

While this article is intended to report a rare sequela following adenoid and tonsil operation, it is also intended as a note of warning against the too frequent and especially the over zealous radical tonsillectomies which are daily being performed. So much has been written especially in the secular periodicals concerning the various ill effects produced upon the child's physical condition by the presence of adenoids in the naso-pharynx, that parents and neighbors now make their own diagnosis without the assistance of the specialist. If one were to believe all that is heard and written about this subject mothers would soon believe that their offspring would become raving idiots if their child's adenoids were not removed. Do not understand that I am lessening the importance of this subject or the necessity of the removal of adenoids when their presence is a menace to the physical well-being of the individual. Experience only can determine this point. The fact that every child 5 or 6 years old has a certain amount of adenoid tissue does not mean that every child should be operated upon. It has been my experience that every young child has adenoid tissue in the naso-pharynx but only when it is obstructive or the producer of a pathologic systemic or local condition should we consider the question of its removal. Because a child is an habitual mouth breather by no means indicates that he or she has a large amount of adenoids. The parent or physician who thinks that the removal of adenoids will remove all symptoms of mouth breathing in every case will be sadly disappointed, for in many of these cases the mouth breathing is due to a short upper lip associated frequently with a high arched palate and in such cases even if every vestage of adenoids is removed, the little patient will still continue to breathe through the mouth. What is needed in most of these cases is the work of the dentist in widening the palatal arch and thus lessen the distance between the nose and the border of the incisor teeth.

After writing this short article instead of completing a bibliography on the subject which would be of very little value, I wrote to fifty members of this Society located in different parts of the country and submitted to them the following question: "Have you ever seen a total or partial paralysis of the soft palate following the removal of faucial tonsils, naso-pharyngeal adenoids or both?"

In this question the word permanent was not used because I had in my mind only the temporary condition not believing that paralysis or paresis would ever be permanent unless there was a very radical surgical mutilation of the soft palate. Leathery infiltrations and restricted movements which are sometimes seen two or three days after tonsil and adenoid operations, are not what I denominate as paresis of the soft palate. In these cases should there be such oedematons infiltrations to as preclude the palate from closing off the naso-pharyngeal space in deglutition, we would have the same symptoms as in paresis or paralysis, but in the former it would be more of a lack of approximation due to the inflammatory thickening of the mucous membrane and muscles. In some of my cases of tonsillectomy in the adult, there has been various degrees of reaction and oedematons infiltratrion but in none of these were seen the symptoms of paresis. Several replies spoke of the temporary restricted movements following these operations but which disappeared in a few days leaving the impression that such was by no means uncommon and was an unimportant sequela. Personally I think that such a sequela is unfortunate and is rather indicative of too much traumatism at the time of operation. The soft palate is an organ of much importance in speech and deglutition and as such is delicate in its mechanism and should require the most gentle treatment in its manipulation.

I believe that all of us are too prone to manipulate roughly in the naso-pharynx at the time of doing an adenoid operation and too frequently do by touch what should be done more by the sense of sight. A swift operation in the naso-pharynx frequently with the palate obscured by blood is by no means an infrequent occurrence among the best of us. The large majority of tonsil and adenoid operations are performed at free clinics and hospitals so that it is natural to suppose many of these are never seen again and it is therefore impossible to know whether or not there was normal convalescence or even what was the final result. The after care of these cases is certainly just as important as any other major surgical operation and they should be kept under observation until all inflammatory reaction has entirely subsided.

From the fifty-five letters sent out I received answers from thirty-nine men. Strange to say there was almost an equal division in the affirmative and negative answers. Sufficient data, however, was obtained to warrant us in considering the question of this paper by no means unimportant in the end results of our every day tonsil and adenoid operation.

These were the replies.

H. Arrowsmith—Brooklyn: "Have never seen any such complication beyond the very temporary disability caused by the reaction following the operation."

W. L. Ballenger—Chicago: "Have seen a few cases of partial and total paralysis of soft palate muscles following tonsil and adenoid operations all recovering in a few days. Regurgition through the nose was present in all cases."

J. E. Barnhill—Indianapolis: "Yes, I have had several. There was the peculiar voice and flow of liquid through the nose on attempt to swallow. This lasted a few days in most cases, several weeks in others."

J. C. Beck—Chicago: "I find that about 10% of cases in private practice have a nasal twang after the operation which might be called paresis. I look upon it more as an infiltration than a paresis."

R. C. Borden—Boston: "Personally I have never had a case of this kind in my private practice but have known of a great many cases of partial paralysis following adenoid operations in the hospital with which I am connected."

J. Payson Clark—Boston: "Have never seen any such case."

Charles N. Cox—Brooklyn: "Have had one such case." private practice. A little girl 8 years old from whom I removed tonsils and adenoids Jan. 28, 1907. Ten days later I noticed a paresis of the soft palate which disappeared in one month."

R. B. Canfield—Ann Arbor, Mich.: "My hospital records show a case of almost complete bilateral paralysis of the soft palate following tonsillectomy in an adult. It persisted for two years gradually improving to complete recovery."

W. L. Culbert—New York: "Have had several cases of this condition but they were very temporary." He aslo speaks of a case of a vocalist now under treatment where some trouble of this kind was experienced after a tonsillectomy.

C. G. Coakley—New York: "No, only restriction of movements which I do not consider as paresis."

E. P. Emerson—Boston: "Have seen several cases of temporary paralysis of the palate in tonsil and adenoid operations but none were permanent."

T. J. Gallaher—Denver: "Have seen slight interference with the function of the soft palate in swallowing but this was due to swelling of the parts."

Charles P. Grayson—Philadelphia: "Have seen no such cases."

Thos. J. Harris—New York: "No."

Lee M. Hurd—New York: "Have seen a number of cases wherein under the present rage of operation the soft palate has been so much deformed that what remained was so rigid as to appear immobile."

O. Joachim—New Orleans: "No."

D. B. Kyle—Philadelphia: "No."

P. D. Kerrison—New York: "No."

M. D. Lederman—New York: "Can recall two cases in my own practice and have seen one other case operated upon by some one else."

Robert Levy—Denver: "Following adenoid operations where unusual traction was made upon the soft palate, have seen patients with faulty articulation and where liquids come through the nose for a few days after the operation. This I presume might be termed traumatic paralysis."

H. W. Loeb—St. Louis: "No."

S. H. Lutz—Brooklyn: "Have seen a few cases wherein undue traumatism was unskillfully produced."

G. Hudson MaKuen—Philadelphia: "Am unable to say just how many cases of insufficiency of the palate have followed the tonsil and adenoid operation, but I agree with you entirely that the condition is by no means an unusual one. I recall one case of almost complete paralysis of the soft palate following a mere digital examination of the vault of the pharynx and this continued for several years."

R. C. Myles—New York: "After the enucleation of imbedded faucial tonsils a condition resembling partial paralysis frequently occurs."

J. W. Murphy—Cincinnatti: "In my experience where large tonsils have been removed and the tonsils extended well up into the soft palate, quite frequently a temporary paralysis has followed this operation. We have had several cases which lasted two or three weeks but eventually completely recovered."

James F. McKernon—New York: "No."

Seymour Oppenheimer—New York: "I have seen a number of cases of paralysis of the soft palate following some of our dissection methods of tonsillectomy. These hav only been transient."

Wendall C. Phillips—New York: "No."

G. Sluder—St. Louis: "No."

Otto Stein—Chicago: "No."

W. E. Sauer—St. Louis: "No."

Harmon Smith—New York: "No."

B. R. Shurley—Detroit: "Have had one case of paralysis of soft palate which persisted for several days."

E. Terry Smith—Hartford, Conn. "No."

Geo. C. Stout—Philadelphia: "My records show three or four such cases."

J. A. Stucky—Lekington, Ky.: "Have had one case of my own in which there was a very large submerged tonsil which after removal by dull dissection and the snare, neither anterior nor posterior pillar being injured and after the removal of an enormous adenoid in same case there was paralysis of the soft palate which continued for six weeks interfering considerably with the patient phonation and especially that of deglutition. Have seen several other such cases in consultation."

C. A. Thigpen—Montgomery, Ala.: "No."

A. P. Voislawsky—New York: "No."

Geo. B. Wood—Philadelphia: "Have had one case several years ago. The condition only lasted for a few days and was due I thought to the stretching of the soft palate by the finger in the vault."

S. Yankauer—New York: "No."

R. McKinney—Memphis, Tenn.: "Have seen only two cases of this character. One occurring in a child six years of age and the other in a young man 22. The child had been operated upon several months previous for tonsils and adenoids. Four days before the writer saw him the child began talking through his nase. Examination showed the velum palati was paralyzed. The boy was kept under observation for some time but his speech did not return to normal. Have not seen him for two years so do not know the final result. The other case was a young man studying vocal and having a baritone voice. A tonsillectomy was done in another city. His voice has lost its resonance and is changed in quality immediately after operation. Examination however showed this condition to be due to a laceration of the parts at the time of the operation.

Grand Opera House, Atlanta, Ga.

